# Role of Protein Kinase C and Nox2-Derived Reactive Oxygen Species Formation in the Activation and Maturation of Dendritic Cells by Phorbol Ester and Lipopolysaccharide

**DOI:** 10.1155/2017/4157213

**Published:** 2017-03-28

**Authors:** Judith Stein, Sebastian Steven, Matthias Bros, Stephan Sudowe, Michael Hausding, Matthias Oelze, Thomas Münzel, Stephan Grabbe, Angelika Reske-Kunz, Andreas Daiber

**Affiliations:** ^1^Department of Dermatology, Medical Center of the Johannes Gutenberg University, Mainz, Germany; ^2^Center for Cardiology/Cardiology 1, Laboratory of Molecular Cardiology, Medical Center of the Johannes Gutenberg University, Mainz, Germany; ^3^Center for Thrombosis and Hemostasis (CTH), Medical Center of the Johannes Gutenberg University, Mainz, Germany

## Abstract

*Aims*. Activation/maturation of dendritic cells (DCs) plays a central role in adaptive immune responses by antigen processing and (cross-) activation of T cells. There is ongoing discussion on the role of reactive oxygen species (ROS) in these processes and with the present study we investigated this enigmatic pathway.* Methods and Results*. DCs were cultured from precursors in the bone marrow of mice (BM-DCs) and analyzed for ROS formation, maturation, and T cell stimulatory capacity upon stimulation with phorbol ester (PDBu) and lipopolysaccharide (LPS). LPS stimulation of BM-DCs caused maturation with moderate intracellular ROS formation, whereas PDBu treatment resulted in maturation with significant ROS formation. The NADPH oxidase inhibitors apocynin/VAS2870 and genetic gp91phox deletion both decreased the ROS signal in PDBu-stimulated BM-DCs without affecting maturation and T cell stimulatory capacity of BM-DCs. In contrast, the protein kinase C inhibitors chelerythrine/Gö6983 decreased PDBu-stimulated ROS formation in BM-DCs as well as maturation.* Conclusion*. Obviously Nox2-dependent ROS formation in BM-DCs is not always required for their maturation or T cell stimulatory potential. PDBu/LPS-triggered BM-DC maturation rather relies on phosphorylation cascades. Our results question the role of oxidative stress as an essential “danger signal” for BM-DC activation, although we cannot exclude contribution by other ROS sources.

## 1. Introduction

Immune cells provide protection of the organism from invading microbial pathogens. The immune system with its various cell types can be divided into innate immune system (e.g., PMN or NK cells) and adaptive immune response (e.g., lymphocytes). Dendritic cells (DCs) serve as a messenger between adaptive and innate immune response [1]. A key event in the adaptive immune response consists of the priming or sensitization of resting T cells, which is a prerequisite for any antigen-induced cellular adaptive immune response. T cell activation largely depends on efficient presentation of antigen fragments by antigen-presenting cells such as DCs. The latter are located in tissues with close contact to the external environment like skin (Langerhans cells) or epithelium of lungs and intestines. DC-dependent activation of T cells also contributes to the development of arterial hypertension [2]. This underlines the clinical relevance of DC activation/maturation for cardiovascular disease. It is known that platelet activation during the course of vascular injury or bleeding and subsequent CD40L release provides a “danger signal” that activates DCs at the site of blood vessel damage and thus facilitates the induction of local T cell-mediated immune responses. In addition, other “danger signals” such as bacterial or fungal endotoxins (e.g., lipopolysaccharide [LPS] or zymosan A), tissue injury markers (e.g., uric acid, hyaluronic acid), immune activation (e.g., CD40L, inflammatory cytokines), and even angiotensin II cause potent activation/maturation of DC [2, 3]. Therefore, an inflammatory and thrombotic environment represents a major trigger for DC activation, maturation, and infiltration, and subsequent immune responses leading to atherothrombotic events in the vasculature.

In addition to the above-described classical “danger signals,” DCs may also become activated by oxidative stress, either via direct immunomodulatory actions of ROS on proteins in immune cells or on mediators of inflammation (e.g., oxLDL, isoketals, and other lipid peroxidation products) [4–11]. In support of this notion, it was shown that oxidative stress in the bone marrow upregulates TNF*α* leading to activation of DCs [12] and that vascular oxidative stress leads to DC activation, adhesion, and transmigration [13]. However, cigarette smoke-induced oxidative stress suppressed the generation of key cytokines by maturing DCs through the activation of ERK-dependent pathways [14]. In turn, ROS have been demonstrated to activate NADPH oxidases in a protein kinase C-dependent fashion [15] opening the opportunity to activate the DC NADPH oxidase (Nox2 isoform). Several publications ascribe an important role of Nox2-derived ROS in antigen processing and presentation by controlling the pH of the phagosome [16–18]. Protein kinase C (isoform *α*) not only is an essential activator of Nox2 activity but also regulates cytokine production of DCs in a MyD88- and toll-like receptor- (TLR-) dependent fashion [19].

With the present study we sought to investigate the role of protein kinase C and Nox2-derived ROS in DC maturation and T cell stimulatory capacity.

## 2. Materials and Methods

### 2.1. Animals and Treatment Protocol

C57BL/6, BALB/cJ, and* gp91*^*phox*^ knockout mice were obtained from the Translational Animal Research Center of the University Medical Center Mainz and maintained under pathogen-free conditions on a standard diet.

Hemizygous *gp*91^*phox*−/*y*^ mice (Nox2 knockout (#2365), C57BL/6 background, Jackson Laboratories, Bar Harbor, ME) were generated as described previously [20] and C57BL/6 mice were used as the corresponding wild type controls. Mice were sacrificed under isoflurane anesthesia and femurs and tibias were recovered for generation of BM-DCs. Animal treatment was in accordance with the Guide for the Care and Use of Laboratory Animals as adopted and promulgated by the US National Institutes of Health and was approved by the Ethics Commission according to the German Law on the Protection of Animals (Landesuntersuchungsamt Rheinland-Pfalz, Koblenz, Germany: #23 177-07/G 10-1-054, #23 170-07/G 07-1-023).

### 2.2. Bone Marrow-Derived DCs

BM-DCs were generated as first described by Scheicher et al. [21] and modified by Bros et al. [22]. Shortly, bone marrow cells (2 × 10^6^ cells/10 ml) were cultured in IMDM supplemented with 5% heat-inactivated FCS (Gibco, Paisley, UK), 2 mM L-glutamine (Roth, Karlsruhe, Germany), 100 U/ml penicillin, 100 *μ*g/ml streptomycin (both PAA, Pasching, Austria), 50 *μ*M *β*-mercaptoethanol (Roth), and 5% GM-CSF-containing cell culture supernatant derived from X63.Ag8-653 myeloma cells stably transfected with a murine GM-CSF expression construct [23] on bacterial dishes (Greiner Bio-One, Frickenhausen, Germany). BM-DC culture medium was replenished on day 3 and day 6 of culture. On day 7, immature BM-DCs were harvested, centrifuged, reseeded in 6-well nontissue culture treated plates (1 × 10^6^ cells/2 ml), and cultured for another 24 hours.

### 2.3. Detection of Extracellular and Intracellular ROS Formation of Cultured BM-DCs

Oxidative burst of cultured BM-DCs (24-well, 1 × 10^6^ cells/well, day 8) was induced by phorbol ester dibutyrate (PDBu, 10 *μ*M) (Sigma-Aldrich, Steinheim, Germany) or LPS (50 *μ*g/ml) (Calbiochem) using L-012- (100 *μ*M-) enhanced chemiluminescence (ECL) in PBS (1 mM Ca^2+^/Mg^2+^) on a Centro plate reader (Berthold Technology, Bad Wildbad, Germany). In some experiments, inhibitors of NADPH oxidase, apocynin (150 or 300 *μ*M, Sigma-Aldrich) or VAS2870 (20 *μ*M, Sigma-Aldrich), or the protein kinase C inhibitors chelerythrine (10, 50, and 100 *μ*M, Sigma-Aldrich) or Gö6983 (0.1, 1, 10 *μ*M, Sigma-Aldrich) were added to the cells 20 min before addition of the L-012 dye and stimulation with PDBu or LPS. ROS formation by BM-DCs (24-well, 2 × 10^5^ cells/well) was also tested by dichlorofluorescein-diacetate (DCF-DA, 20 *μ*M) or dihydroethidium (DHE, 1 *μ*M) fluorescence using a Mithras 2 fluorescence plate reader for DCF-DA (Berthold Techn., Bad Wildbad, Germany; filters for excitation: 485 ± 10 nm, emission: 535 ± 10 nm) and fluorescence microscopy for DHE (Axiovert, Zeiss). DCF-DA was added with the inhibitor to the cells 20 min before stimulation with PDBu or LPS.

### 2.4. Detection of Intracellular ROS Generation by Flow Cytometry (FACS)

On day 8 of culture, an aliquot of BM-DCs was incubated with the dye CM-DCF-DA (5 *μ*M/1 × 10^6^/ml) (Thermo Fisher Scientific, Waltham, MA) for 30 min, while another aliquot was left untreated. Cells were stimulated with LPS (1.5 *μ*g/ml), various concentrations of PDBu (0.1 *μ*M, 1 *μ*M, or 10 *μ*M), PMA (50 ng/ml, Sigma-Aldrich), zymosan A (50 *μ*g/ml, life technologies), lipoteichoic acid (10 *μ*g/ml, Sigma-Aldrich), activating antiCD40 Ab (10 *μ*g/ml), mouse recombinant soluble CD40L (0.5 *μ*g/ml, ImmunoTools, Frisoythe, Germany), TNF-*α*/IL-1*β* (10 ng/mg each, ImmunoTools), or remained unstimulated as a control for the time points indicated. Cells were harvested and washed in staining buffer (PBS/2% FCS). To avoid Fc receptor-mediated nonspecific binding of antibodies (Ab), cells were incubated for 15 min on ice with a rat anti-mouse CD16/CD32 Ab (clone 2.4G2, purified from hybridoma supernatant). Cell surface was stained with a phycoerythrin- (PE-) conjugated Ab recognizing CD11c (clone N418, Miltenyi Biotec, Bergisch-Gladbach, Germany) and a phycoerythrin-cyanine 5- (PE-Cy5-) conjugated Ab recognizing MHCII (clone M5/114, eBioscience, San Diego, CA). Suitable isotype control Abs were used. Flow cytometry measurement was performed using a FACS Canto II flow cytometer (BD Biosciences) and analyzed using FlowJo software. In particular, DCs were identified by the expression of the surface marker CD11c (named CD11c^+^ cells), and these were subsequently subdivided into MHCII^high^ expressing DCs and MHC^low^ expressing DCs as indicated in suppl. Figure 1S available online at https://doi.org/10.1155/2017/4157213. ROS generation was analyzed in both subpopulations.

### 2.5. Detection of Maturation Using FACS

Stimulated and unstimulated BM-DCs were harvested and Fc-receptor block was performed as described above. Cell surface was stained with the following Abs: fluorescein isothiocyanate- (FITC-) conjugated anti-CD11c (clone N418, Miltenyi Biotec), PE-conjugated anti-CD86 (clone GL1, eBioscience), and PE-Cy5-conjugated anti-MHCII (clone M5/114, eBioscience). Cells were fixed with 0.7% paraformaldehyde (Merck, Darmstadt, Germany) in PBS and subjected to FACS analysis as described above. As described above, DCs were defined as CD11c^+^ cells, and expression of MHCII^high^ and CD86^high^ characterized these cells as mature DCs as indicated in suppl. Figure 2S.

### 2.6. Allogeneic T Cell Stimulation Assays

Splenic BALB/cJ T cells (3 × 10^5^) enriched by nylon wool adherence as described [24] were cocultured with serially diluted C57BL/6 or *gp*91^*phox*−/*y*^ BM-DCs (start concentration: 5 × 10^4^) in triplicates in 200 *μ*l culture medium without GM-CSF in 96-well flat-bottom plates for 4 days. Proliferation was assessed by measuring the genomic incorporation of [^3^H] thymidine (0.25 *μ*Ci/well), which was added for the last 16 h of the culture. Cells were harvested onto glass fiber filters and retained radioactivity was measured in a liquid scintillation counter (1205 Betaplate, LKB Wallac, Turcu, Finland).

### 2.7. Statistical Analysis

Data are expressed as mean ± SEM. Statistically significant differences were assessed by using one-way ANOVA for comparisons of groups followed by pairwise comparison analysis using Holm-Sidak. *p* values < 0.05 were considered statistically significant (^*∗*^*p* < 0.05, ^*∗∗*^*p* < 0.01, and ^*∗∗∗*^*p* < 0.001). Analysis was performed for most data by using SigmaPlot 12.3 (Systat Software, San Jose, CA) and for chemiluminescence and fluorescence data of ROS formation by employing Prism 6 for Windows, version 6.05, GraphPad Software.

## 3. Results

### 3.1. LPS and the PKC-Activator PDBu Promote BM-DC Maturation, but Only PDBu Increases Intracellular ROS Formation in BM-DCs

**Figure 1:**
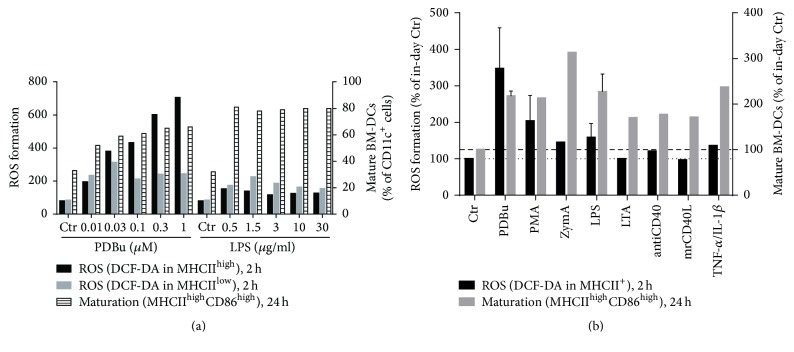
Analysis of ROS formation 2 h and maturation (high MHCII and CD86 expression) 24 h after stimulation of BM-DCs with (a) increasing concentrations of PDBu (0.01–1 *μ*M) or LPS (0.5–30 *μ*g/ml) or (b) PDBu (0.1 *μ*M), PMA (50 ng/ml), zymosan A (ZymA, 50 *μ*g/ml), LPS (1.5 *μ*g/ml), lipoteichoic acid (LTA, 10 *μ*g/ml), antiCD40 (10 *μ*g/ml), mrCD40L (0.5 *μ*g/ml) or TNF-*α*/IL-1*β* (10 ng/ml each) by flow cytometry. Representative data of one single experiment are shown (a) and mean ± SEM of *n* = 1–3 experiments (b).

**Figure 2:**
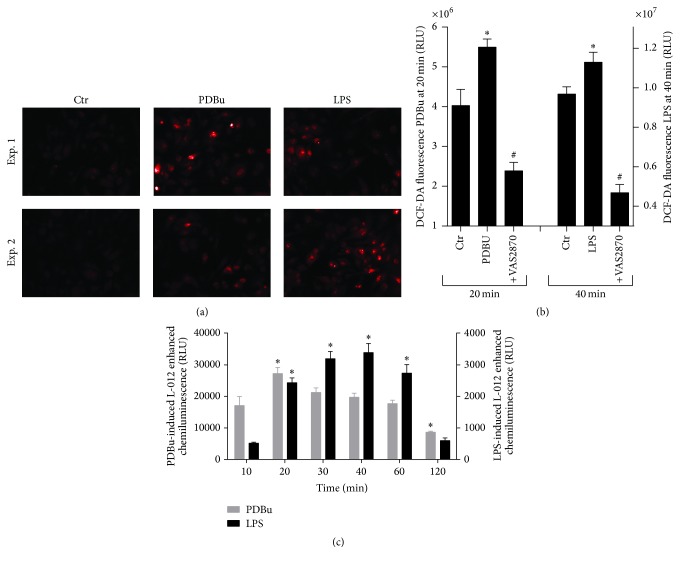
Treatment of BM-DCs (2 × 10^5^ cells/well) with PDBu (10 *μ*M) and analysis of the formation of intra- and extracellular ROS measured by (a) DHE (1 *μ*M) fluorescence microscopy 20 min after addition of PDBu (10 *μ*M) or LPS (50 *μ*g/ml) (magnification 200x), (b) DCF-DA (20 *μ*M) fluorescence in a plate reader 20 min after addition of PDBu, or 40 min after addition LPS in the presence or absence of the NADPH oxidase inhibitor VAS2870 (20 *μ*M). (c) Kinetics of ROS formation were measured by L-012 (100 *μ*M) ECL upon addition of PDBu or LPS. Representative images of 2 independent experiments (a) and mean ± SEM of 8 (b) and 8 (c) experiments are shown. ^*∗*^*p* < 0.05 versus Ctr (w/o PDBu or LPS) or ^#^*p* < 0.05 versus stimulated (with PDBu or LPS) (b) and ^*∗*^*p* < 0.05 versus time point 10 min (c).

In a set of pilot experiments, we observed that the phorbol ester PDBu induced a concentration-dependent increase in ROS formation in MHCII^high^ cells, whereas maturation was augmented by the lowest concentration of PDBu and showed only marginal further increase at higher concentrations of PDBu (Figure 1(a)). In contrast, the endotoxin LPS at all concentrations employed increased the maturation of BM-DCs to a similar extent but led to a less pronounced augmentation of the ROS formation rate as compared to PDBu (Figure 1(a)). Puzzled by this obvious dissociation between ROS formation and maturation in BM-DCs we tested a set of different stimulators of BM-DC maturation. We found that only phorbol esters induced ROS formation and maturation, whereas all other classical stimulators induced maturation without significant ROS formation (Figure 1(b)). In order to exclude artificial ROS signals by FACS analysis, we tested other ROS detection methods. Staining of cultured BM-DCs with the fluorescence dyes dihydroethidium (DHE) or dihydrodichlorofluorescein diacetate (DCF-DA) revealed a quite similar increase in intracellular ROS formation upon stimulation of BM-DCs with PDBu or LPS (Figures 2(a) and 2(b)). Due to a high background signal, the increase in ROS formation upon stimulation with PDBu or LPS was less obvious with DCF-DA but was significantly suppressed by the NADPH oxidase inhibitor VAS2870. Using the luminol analogue and chemiluminescence dye L-012 we established a time course for PDBu- and LPS-induced extracellular ROS formation by BM-DCs with maxima at 20 and 40 min, respectively (Figure 2(c)). Maximal ROS formation revealed by L-012 ECL was approximately 10-fold more pronounced with PDBu stimulation as compared with the LPS-treated group. Therefore, the overall ROS signal induced by the two stimulators largely depends on the site of ROS measurement and the employed dye.

**Figure 3:**
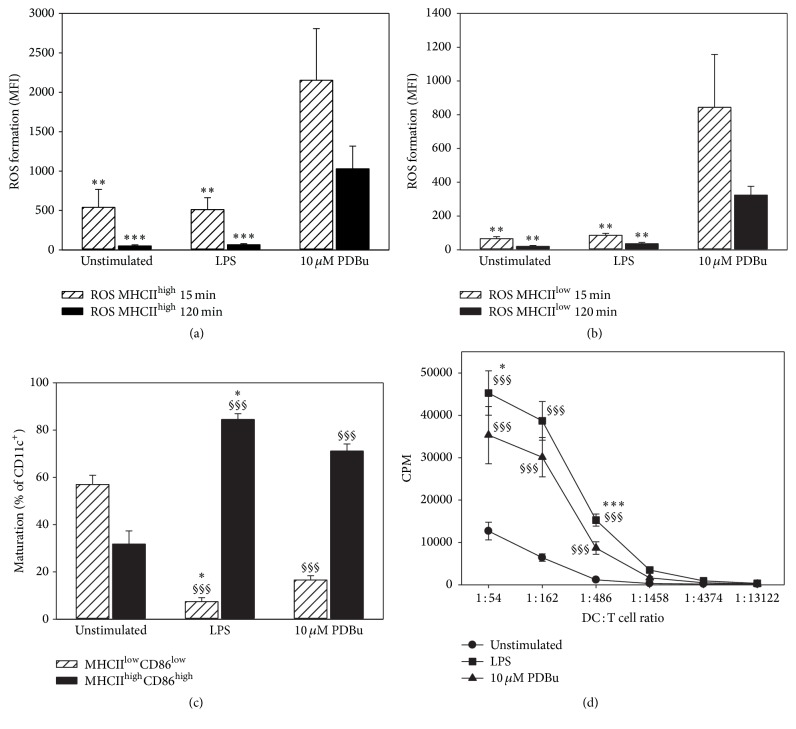
Analysis of intracellular ROS formation upon activation of BM-DCs, of maturation at 24 h after stimulation, and of their T cell stimulatory capacity. (a) 1 × 10^6^ BM-DCs were stimulated with LPS (1.5 *μ*g/ml) or PDBu (10 *μ*M). ROS formation (a) in MHCII^high^ and (b) MHCII^low^ expressing CD11c^+^ cells was analyzed by flow cytometry after 15 or 120 min. (c) Maturation was characterized by high MHCII and CD86 expression after 24 h using flow cytometry. (d) T cell stimulatory capacity was compared in a proliferation assay with a constant number of T cells (3 × 10^5^) cocultured with decreasing numbers of differentially stimulated BM-DCs, and on day 4 proliferation of T cells was measured by incorporation of [^3^H] thymidine. Data are mean ± SEM of (a) 4-5, (b) 4-5, (c) 5, and (d) 3 experiments. ^*∗∗∗*^*p* < 0.001; ^*∗∗*^*p* < 0.01 versus PDBu (a, b); ^§§§^*p* < 0.001 versus unstimulated; ^*∗*^*p* < 0.05; ^*∗∗∗*^*p* < 0.001 versus PDBu (c, d).

More detailed FACS analysis confirmed these initial findings on the dissociation between ROS formation and maturation of BM-DCs. Only PDBu, but not LPS, resulted in a significant increase in ROS formation by BM-DCs at 15 and 120 min (Figures 3(a) and 3(b)), whereas both stimuli increased the maturation at 24 h after stimulation to a similar degree (Figure 3(c)). Furthermore, T cell stimulatory capacity of BM-DCs was increased by PDBu and LPS (Figure 3(d)).

### 3.2. PDBu-Induced Maturation of BM-DCs Can Be Blocked by PKC-Inhibition and Does Not Depend on Nox2-Derived ROS Formation

**Figure 4:**
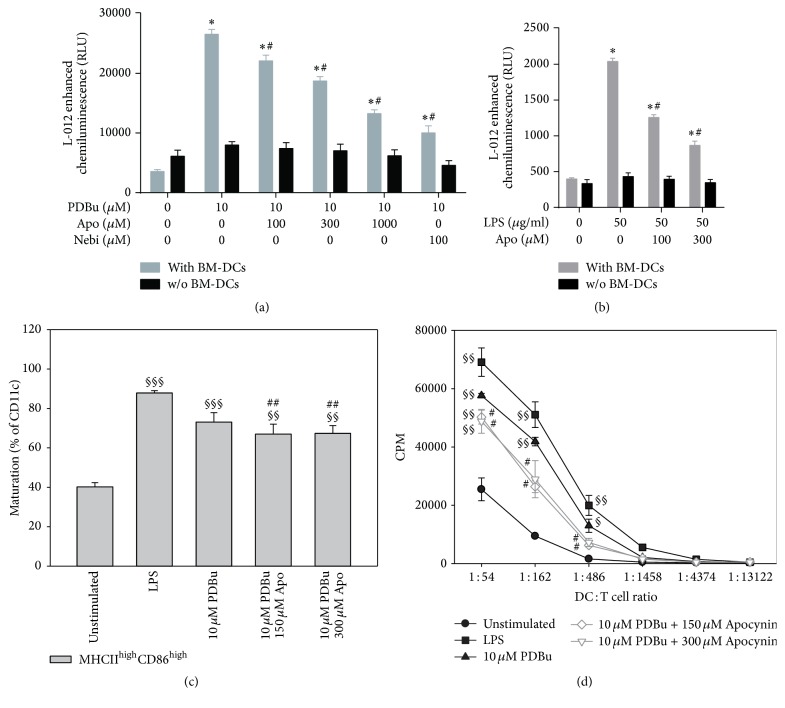
Analysis of the effects of apocynin treatment on the formation of ROS, maturation, and the T cell stimulatory capacity of BM-DCs upon activation by PDBu or LPS. (a) BM-DCs (1 × 10^6^ cells/well) were stimulated with PDBu (10 *μ*M) and additionally treated with different concentrations of apocynin (100–1000 *μ*M) or with the potent inhibitor of Nox2 activity nebivolol (100 *μ*M), and ROS generation was measured by L-012 chemiluminescence at 10 min after stimulation. (b) ROS formation after stimulation of BM-DCs (1 × 10^6^ cells/well) with LPS (50 *μ*g/ml) and cotreatment with apocynin (100–300 *μ*M) was measured by L-012 chemiluminescence at 40 min after stimulation. (c) Maturation of BM-DCs (expression of MHCII and CD86 at high level) was analyzed after stimulation with LPS (1.5 *μ*g/ml) or PDBu (10 *μ*M) and treatment with apocynin (150–300 *μ*M) after 24 h via flow cytometry. (d) T cell stimulatory capacity after apocynin treatment was compared in a proliferation assay (see legend to Figure 3). Data are mean ± SEM of 8 (a, b) and 3 (c) experiments. (d) One representative experiment of two is shown. (a, b) ^*∗*^*p* < 0.05 versus Ctr (w/o PDBu or LPS); ^#^*p* < 0.05 versus PDBu or LPS treated sample; (c) ^§§§^*p* < 0.001; ^§§^*p* < 0.01 versus unstimulated; ^##^*p* < 0.01 versus LPS. (d) ^§§^*p* < 0.01; ^§^*p* < 0.05 versus unstimulated; ^#^*p* < 0.05 versus PDBu.

Since previous studies identified the NADPH oxidase system (mainly the Nox2 isoform) as a potent source of ROS in activated BM-DCs, we tested the effect of the pharmacological inhibitor of NADPH oxidase-derived ROS formation, apocynin, on the ROS signal, and maturation of BM-DCs. We established a concentration-dependent inhibition of BM-DC-derived extracellular ROS formation by apocynin and the known Nox2 inhibitor nebivolol [25, 26] (Figure 4(a)). Also, LPS-induced extracellular ROS formation by BM-DCs was decreased by apocynin in a dose-dependent fashion (Figure 4(b)). In contrast, apocynin only caused a minor inhibition of maturation (Figure 4(c)) but a more pronounced loss of T cell stimulatory capacity (Figure 4(d)) of PDBu-stimulated BM-DCs. The latter would be compatible with numerous reports on a role of Nox2 in antigen presentation by BM-DCs that is based on pH modulation in the phagosome [16–18].

**Figure 5:**
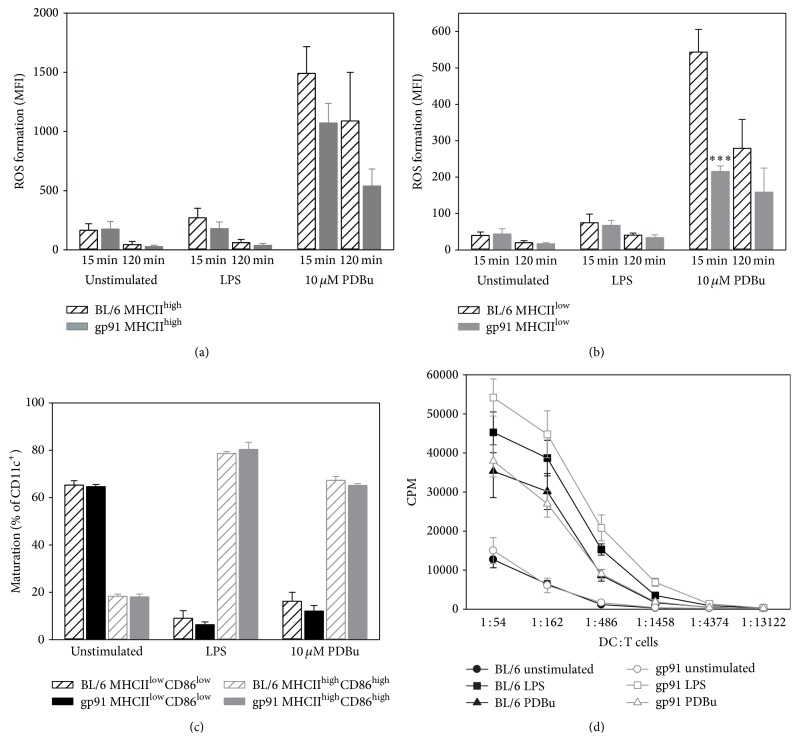
Analysis of the effects of genetic knock out of the gp91^phox^ subunit of the phagocytic NADPH oxidase on ROS formation, maturation, and T cell stimulatory capacity of PDBu- or LPS-activated BM-DCs. BM-DCs of control mice (C57BL/6) and gp91^phox−/y^ mice were stimulated with LPS (1.5 *μ*g/ml) or PDBu (10 *μ*M). ROS formation was measured after 15 and 120 min in MHCII^high^ (a) or MHCII^low^ (b) expressing CD11c^+^ cells. (c) Analysis of maturation represented by high expression of MHCII and CD86 24 h after stimulation. (d) Analysis of T cell stimulatory capacity on day 4. Data are mean ± SEM of (a) 3, (b) 3, (c) 3, and (d) 3 experiments. ^*∗∗∗*^*p* < 0.001 versus C57BL/6 PDBu at 15 min.

Since apocynin, especially at higher concentrations, has direct antioxidant properties [27], we also tested genetic deletion of the Nox2 subunit gp91phox in order to characterize the role of Nox2-derived ROS in BM-DC maturation and T cell stimulatory capacity. Although we observed a significant decrease in ROS formation by PDBu-stimulated BM-DCs of gp91phox-deficient as compared with control mice (Figures 5(a) and 5(b)), neither maturation nor T cell stimulatory capacity was significantly impaired in gp91phox-deficient animals upon PDBu stimulation (Figures 5(c) and 5(d)). However, since PDBu-induced ROS formation was not completely absent in BM-DCs without gp91phox, we cannot exclude contribution of other sources of ROS (e.g., mitochondria or other Nox isoforms) to BM-DC maturation and T cell stimulatory capacity.

**Figure 6:**
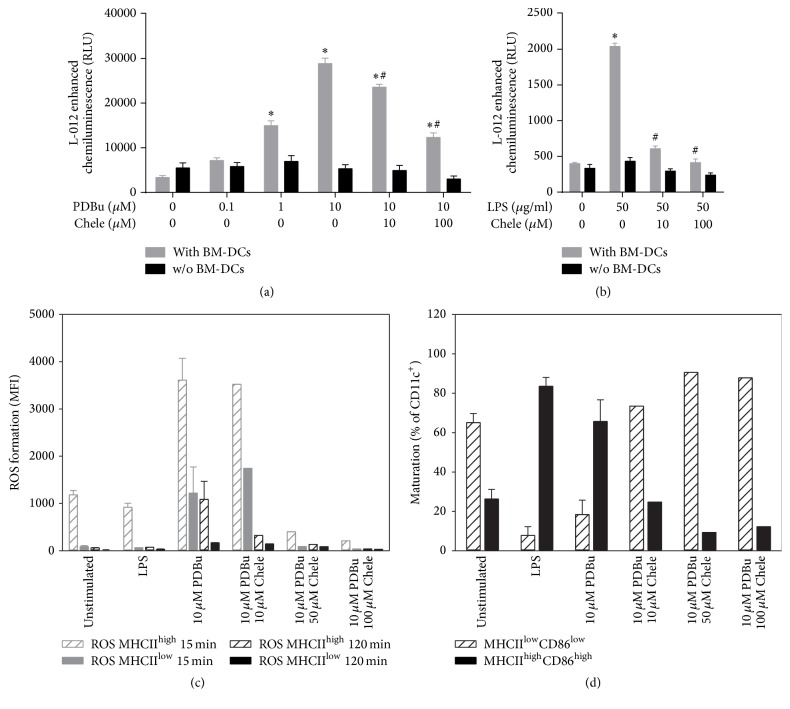
Analysis of the effects of chelerythrine treatment on the formation of ROS and maturation of BM-DCs upon activation by PDBu or LPS. (a) BM-DCs (1 × 10^6^ cells/well, 24-well plate) were stimulated with PDBu (0.1, 1, and 10 *μ*M) and additionally treated with different concentrations of chelerythrine (10 and 100 *μ*M), and ROS generation was measured by L-012 chemiluminescence at 20 min after stimulation. (b) ROS formation after stimulation of BM-DCs (1 × 10^6^ cells/well, 24-well plate) with LPS (50 *μ*g/ml) and cotreatment with chelerythrine (10 and 100 *μ*M) was measured by L-012 chemiluminescence at 40 min after stimulation. Analysis of ROS generation (c) and maturation (d) after stimulation with LPS (1.5 *μ*g/ml) or PDBu (10 *μ*M) and cotreatment with chelerythrine (10–100 *μ*M) using flow cytometry. Synthesis of 2 experiments (controls were merged) with different chelerythrine concentrations. Data are mean ± SEM of 8 (a, b) and 1-2 (c, d) experiments. ^*∗*^*p* < 0.05 versus Ctr (w/o PDBu or LPS); ^#^*p* < 0.05 versus PDBu- or LPS-treated sample.

**Figure 7:**
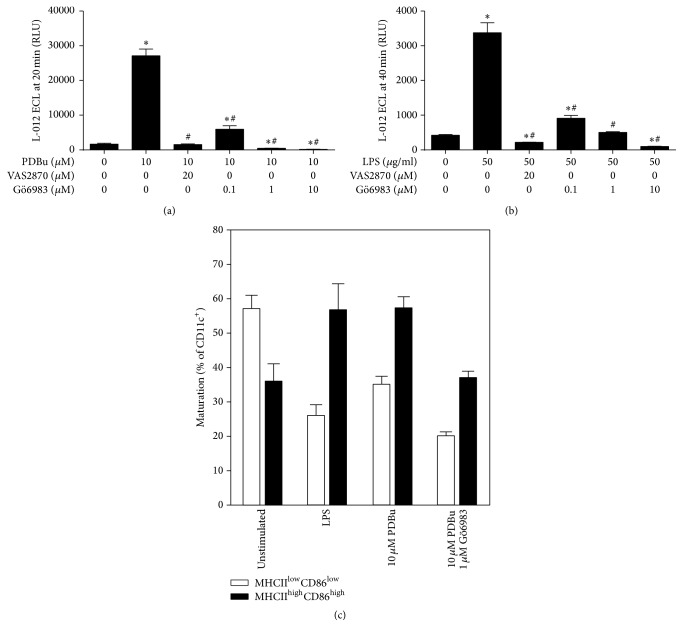
Analysis of the effects of the treatment with the NADPH oxidase inhibitor VAS2870 and the PKC inhibitor Gö6983 on the formation of ROS and maturation of BM-DCs upon activation by PDBu or LPS. (a) BM-DCs (2 × 10^5^ cells/well, 96-well plate) were stimulated with PDBu and additionally treated with VAS2870 and Gö6983, and ROS generation was measured by L-012 (100 *μ*M) chemiluminescence at 20 min after stimulation. (b) ROS formation after stimulation of BM-DCs (2 × 10^5^ cells/well, 96-well plate) with LPS and cotreatment with VAS2870 and Gö6983 was measured by L-012 chemiluminescence at 40 min after stimulation. (c) Analysis of maturation of BM-DCs (1 × 10^6^ cells/well, 24-well plate) after stimulation with LPS (1.5 *μ*g/ml) or PDBu (10 *μ*M) and cotreatment with Gö6983 using flow cytometry. Data are mean ± SEM of 8 (a, b) and 2 (c) experiments. ^*∗*^*p* < 0.05 versus Ctr (w/o PDBu or LPS); ^#^*p* < 0.05 versus PDBu- or LPS-treated sample.

Nox2 activation is mainly mediated by PKC [28], which in turn was reported to essentially contribute to DC cytokine production [19]. We therefore tested the effect of the PKC inhibitor chelerythrine on BM-DC-derived ROS formation and maturation. Chelerythrine induced a concentration-dependent inhibition of PDBu- and LPS-induced BM-DC-derived extracellular ROS formation (Figures 6(a) and 6(b)). Also PDBu-induced intracellular ROS formation by BM-DCs (measured by FACS analysis) was decreased by chelerythrine in a dose-dependent fashion (Figure 6(c)). LPS-triggered ROS formation was clearly less pronounced as compared to the PDBu-treated group. PDBu-triggered BM-DC maturation was completely abolished by chelerythrine (Figure 6(d)). In an independent experiment we showed that the Nox2 inhibitor VAS2870 as well as the alternative PKC inhibitor Gö6983 inhibited the PDBu or LPS-stimulated extracellular ROS signal in BM-DCs (Figures 7(a) and 7(b)). As shown for chelerythrine, also Gö6983 decreased the PDBu-triggered BM-DC maturation (Figure 7(c)).

## 4. Discussion

The results of the present study demonstrate that the phorbol ester PDBu like LPS, which are classical stimulators for oxidative burst in PMN, trigger BM-DC maturation and T cell stimulatory capacity. We aimed to identify the role of oxidative stress in BM-DC maturation and found that PDBu and to a minor extent also LPS induced intracellular ROS formation in BM-DCs. Interestingly, inhibition of NADPH-oxidase by apocynin, VAS2870, or genetic knock-out of Nox2 subunit gp91phox had only minor or no effect on BM-DC maturation and T cell stimulatory capacity. However, inhibition of protein kinase C by chelerythrine or Gö6983 inhibited both PDBu-induced maturation and ROS formation. Taken together, we here provide evidence against an essential role of intracellular Nox2-derived ROS formation in BM-DC maturation by PDBu and LPS.

The phagocyte NADPH oxidase Nox2 is a well-known player in host defense of the innate immune system, and there is increasing evidence for a role of Nox2 in adaptive immunity [29–31]. Patients suffering from chronic granulomatous disease (CGD) have a higher susceptibility to autoimmune diseases like lupus erythematosus or rheumatoid arthritis [32, 33]. Maturation of DCs is part of the pathogenesis of such autoimmune diseases [34]. Nox2-deficient mice crossed onto a lupus-prone genetic background (MRL.Fas^lpr^) develop higher antibody titer and a more severe clinical picture of the disease than control mice [35]. Furthermore, Nox2 is involved in antigen presentation by BM-DCs, which is based on pH modulation in the phagosome [16–18].

However, the role of Nox2-derived ROS in autoimmune disease is not clear until now and the literature data reflect a quite contradictory picture of the role of ROS formation in DC maturation. It was shown that ROS-low DCs displayed a more pronounced response to TLR agonists like LPS and zymosan, followed by accelerated maturation and T cell stimulatory capacity as compared with ROS-high DCs, which showed increased MAPK signaling, adhesion and hydrogen peroxide release indicating their role in immediate microbial targeting [36]. In contrast, liposome-driven ROS formation and chemokine (ERK-dependent)/cytokine (MAPK-dependent) production were all prevented by unspecific antioxidants (TEMPO and ebselen), an inhibitor of flavin-dependent oxidoreductases including NADPH oxidases (diphenyl iodonium) and a MAPK inhibitor (SB203580) [37]. Although representing an unspecific approach, cigarette smoke extract induced ROS formation, production of the chemokines CCL3 and CXCL2, and NF*κ*B activation in a TLR-dependent fashion, all of which were prevented by the antioxidant N-acetylcysteine [38]. Another unspecific approach was based on LPS-triggered ROS formation by DCs and their activation (maturation, cytokine production, and T cell stimulatory potential), all of which were prevented by the antioxidant ebselen [39], however, keeping in mind that this compound is an unspecific inhibitor of thiol-dependent enzymes [40]. Also, treatment with a glutathione ester successfully prevented LPS-induced mitochondrial ROS formation (measured by electron spin resonance spectroscopy) and cytokine production [41]. Mitochondrial ROS formation was demonstrated to be an essential contributor to DC differentiation since the complex I inhibitor rotenone but also catalase in the culture medium decreased DC-derived ROS levels and markers of differentiation, pointing towards an essential role of mitochondrial hydrogen peroxide in this process [41]. This is in line with the observation that mitochondrial ROS can trigger hypoxia-inducible factor 1-dependent reprogramming and switch to glycolysis in various cell types including DC [42, 43].

Previously it was shown that NADPH oxidase-derived ROS did not contribute to DC differentiation, maturation, cytokine production, and induction of T cell proliferation, since DC generated from patients with chronic granulomatous disease (CGD) displayed normal function [44]. Likewise, treatment with a ROS scavenger also had no effect in the hands of these authors. In contrast, NADPH oxidase activation was essential for DC-mediated killing of intracellular* E. coli* [44], which is supported by various cases indicating exacerbated inflammatory reactions upon bacterial infections of CGD patients [45]. In line with this previous observations, our data indicate a minor role of Nox2-derived ROS formation for BM-DC maturation and T cell stimulatory capacity upon activation by PDBu or LPS. Nevertheless, since neither PDBu-induced nor LPS-induced ROS formation was completely absent in BM-DCs without gp91phox, it might be speculated whether other sources of ROS (e.g., mitochondria or other Nox isoforms) contribute to BM-DC maturation and T cell stimulatory capacity. Also, as shown in multiple studies on experimental sepsis (endotoxemia), LPS-dependent activation of immune cells is associated with appreciable levels of ROS formation, which most likely originate from mitochondria and NADPH oxidases [46, 47]. In general, it is also well known that ROS over production in immune cells leads to cell dysfunction and impaired/unregulated immune response and end organ damage [48–51], although in some rare diseases also insufficient ROS production may contribute to uncontrolled inflammatory conditions [52]. In most cases, this implies that even in LPS models ROS may play a role in the activation of immune cells as well as resolution of inflammation, as shown for mitochondrial ROS in LPS-primed bone marrow-derived macrophages [53]. In support of this it was shown in several experimental sepsis models that LPS-induced ROS formation is decreased and survival is improved in endotoxemic gp91phox knockout mice or by pretreatment with apocynin [54–56]. In contrast, in human genetic gp91phox deficiency (like in CGD patients) even exacerbation of LPS-induced inflammation was reported [45].

In conclusion, we provide evidence for an important role of protein kinase C in BM-DC maturation, since PKC blockade by chelerythrine or Gö6983 inhibited PDBu-dependent BM-DC maturation. There are only few reports on PKC stimulatory effects on BM-DC maturation, but it was reported to essentially contribute to DC cytokine production [19].

## 5. Limitations

A major problem in the comparison of the different studies on DC-dependent ROS production and its impact on maturation might be the exact time point of measurement. As long as no kinetics of ROS formation are provided it is hard to assign the signal to a certain source of ROS. Also the fact that FACS analyses are restricted to the measurement of intracellular ROS formation represents a major limitation of the previous studies on effects of ROS on DC maturation and T cell stimulatory capacity.

Moreover, in vivo activation and maturation of DC are multifactorial and antigen/disease specific [3, 57]. Of note, other sources of ROS (e.g., mitochondria) as well as oxidation products generated by ROS (e.g., oxLDL, reactive aldehydes, and isoketals) are known to contribute to DC activation and maturation [6, 9–11]. Likewise, ROS from external Nox2 sources (e.g., other activated immune cells) could trigger BM-DC maturation in vivo. These “external” factors could be missing in our BM-DC cell culture model. In fact, our present data indicate that not all DC activation and maturation require ROS but may solely depend on the toll-like receptor activation and cell signaling pathways such as PKC.

The identification of the specific PKC isoform(s) that contribute to this ROS independent signaling by future experiments would provide important molecular insights in the activation mechanisms of DCs.

## 6. Conclusions and Clinical Implications

Our findings question the concept that Nox2-dependent ROS formation in BM-DCs is required for their maturation or their T cell stimulatory potential (based on the absence of any effect of gp91phox deficiency or the NADPH oxidase inhibitor apocynin). The observed phorbol ester-triggered BM-DC maturation seems to rather depend on phosphorylation cascades than on Nox2-derived ROS formation (based on the observed effects with the PKC inhibitors chelerythrine and Gö6983). However, we cannot exclude that other sources of ROS in DCs (e.g., mitochondria) or ROS coming from external Nox2 sources (e.g., other immune cells) or ROS-generated oxidized DC activators such as lipid oxidation products contribute to BM-DC maturation and their T cell stimulatory potential in vivo. Since DCs provide an essential link between adaptive and innate immune response and confer the priming or sensitization of resting T cells, which is a prerequisite for any antigen-induced cellular adaptive immune response, their activation represents a fundamental mechanism in all kinds of immune responses but also progression of autoimmune diseases and low-grade inflammation triggered cardiovascular, metabolic, and neurodegenerative pathologies. Our results question the role of oxidative stress as an essential “danger signal” for BM-DC activation.

## Supplementary Material

The supplementary material explains the gating strategy for FACS analyses: suppl. Figure 1S “ROS detection” and suppl. Figure 2S “maturation status”.
