# Supplementary material for: Role of Protein Kinase C and Nox2-Derived Reactive Oxygen Species Formation in the Activation and Maturation of Dendritic Cells by Phorbol Ester and Lipopolysaccharide
Source: Oxid Med Cell Longev. 2017 Mar 28;2017:4157213. doi: 10.1155/2017/4157213 (PMC5387830; doi:10.1155/2017/4157213)

Online supplemental figures

Judith Stein et al.

Suppl. Figure 1S

Gating strategy for ROS detection

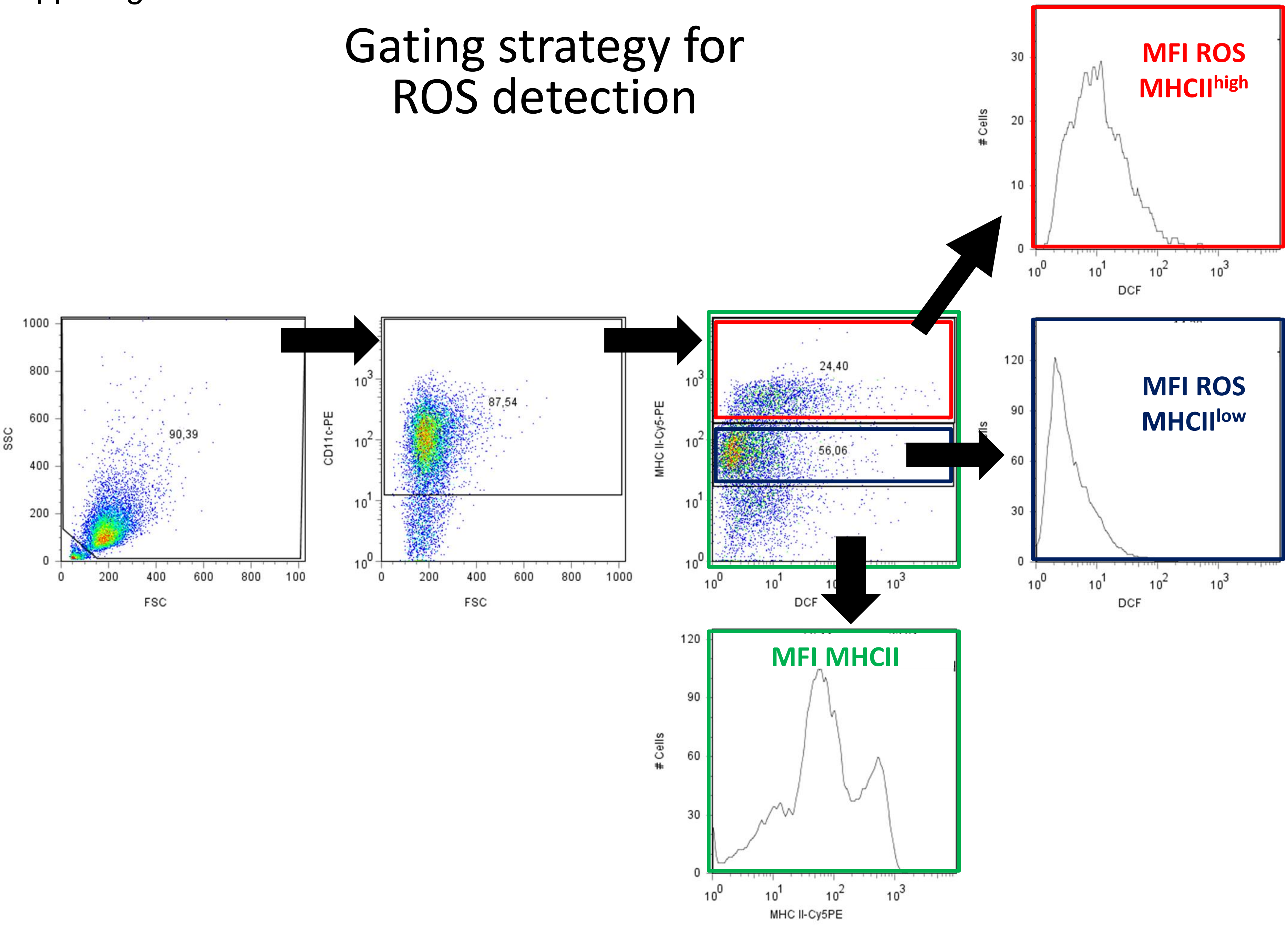

Suppl. Figure 2S

Gating strategy for estimation of maturation status

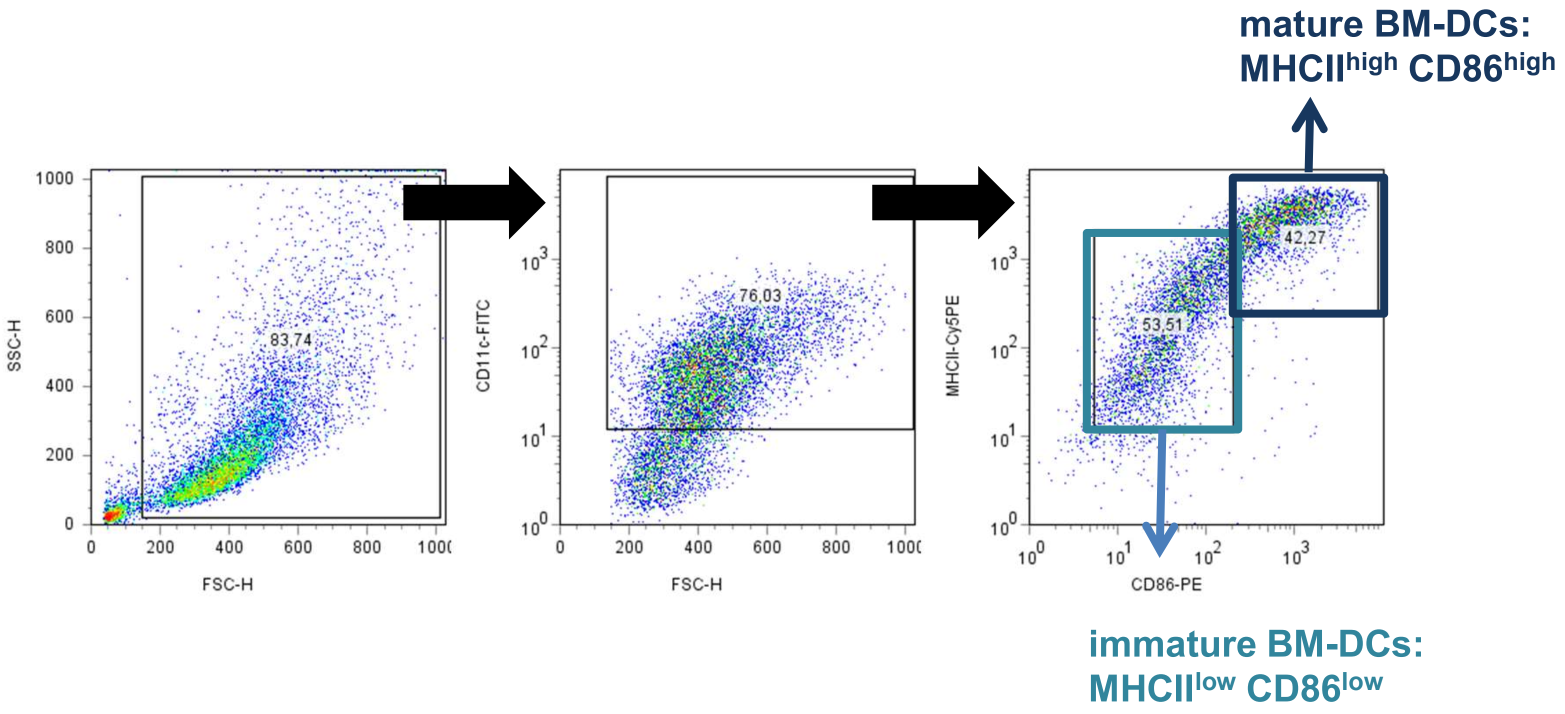

Supplement: Supplementary file 1 — The supplementary material explains the gating strategy for FACS analyses: suppl. Figure 1S “ROS detection” and suppl. Figure 2S “maturation status”. [file 4157213.f1.pdf]
